# The Occupational Hazards of Tattoo Artists: A Scoping Review

**DOI:** 10.7759/cureus.104255

**Published:** 2026-02-25

**Authors:** Eleanor I Barden, Tabitha F Hutchison, Lauri Ann Maitland

**Affiliations:** 1 Osteopathic Medicine, Liberty University College of Osteopathic Medicine, Lynchburg, USA; 2 Medical School, Liberty University College of Osteopathic Medicine, Lynchburg, USA; 3 Family Medicine, Liberty University College of Osteopathic Medicine, Lynchburg, USA

**Keywords:** bloodborne pathogens, chemical exposure, musculoskeletal disorders, needle stick injury, occupational hazards, psychological risk, scoping review, tattoo artists

## Abstract

The art of tattooing involves exposure to physical, chemical, and biological risk factors that parallel procedural medical professions. While risks related to tattoos themselves have been discussed in the context of client safety, no summative literature on occupational risks to artists exists in academic literature today. This scoping review aims to characterize and catalogue the scope of the existing literature on occupational risk hazards faced by tattoo artists.

This scoping review was conducted in accordance with the Joanna Briggs Institute (JBI) methodology for scoping reviews and the Preferred Reporting Items for Systematic reviews and Meta-Analyses extension for Scoping Reviews (PRISMA-ScR) reporting guidelines. An a priori protocol was developed and registered on the Open Science Framework (OSF). After preliminary searches, two independent reviewers selected literature based on the inclusion criteria on occupational risk factors for tattoo artists. These results were tabulated and examined narratively. Results were categorized based on risk type into five categories: musculoskeletal complaints, biological hazards, sharp instrument injury, psychosocial risk, and chemical hazards. The largest number of studies examined biological risks.

This scoping review demonstrates that despite clearly defined occupational hazards, the academic literature addressing these hazards among tattoo artists remains limited and methodologically narrow. The limited existing studies rely on self-reported, cross-sectional designs and focus on biological risks, sharps-related dangers, and musculoskeletal risks, with significantly less attention given to chemical and psychological risks. Future research should prioritize objective and observational methodologies, with consideration made for regionally specific professional requirements.

## Introduction and background

Previous literature has described historical risks associated with tattooing, including infectious complications with viral hepatitis attributable to contaminated equipment and inadequate hygiene practices during periods when single-use needles and standardized infection-control protocols were less consistently implemented; these risks have been substantially reduced with improved infection control standards [1]. 

Dermatologic and neoplastic complications involving tattooed skin, including malignant melanoma as well as bacterial and parasitic infections, have been reported [1-3]. Despite a growing body of literature examining tattoo-related complications, there remain limited studies specifically addressing occupational hazards faced by tattoo artists. 

A precedent in research exists for comparative analysis between tattoo artists and healthcare professionals [4]. One example of this similarity is that tattoo artists have been shown to experience similar patterns of musculoskeletal injury to dentists [5]. Given the parallels between procedural medical professionals and tattoo artists, similar considerations can be made concerning personal protection measures. Tattooing necessitates the exposure of artists to bodily fluids. Examination of biological risks associated with bodily fluid exposure and risk mitigation practices has been formally studied, including administrative standards, infection control procedure and hepatitis B vaccine access [6]. It has been demonstrated that since tattooing requires the artist to hold a needle in their hand for the procedure, there is a risk of inadvertent sharp instrument injury. While this area is understudied, a cross-sectional survey demonstrated a high incidence of contaminated needle injury per artist surveyed [7]. A precedent for examining exposure to chemical agents as risk factors for tattooers has also been set. The paucity of research on occupational chemical exposure highlights the potential for injury and the need for further study, while no specific chemical risks have yet been identified [8]. Psychosocial risk considerations of the profession have been examined in a historical and narrative sense due to the complex social history of the profession [9].

This scoping review aims to comprehensively summarize and integrate the research on occupational risks to tattoo artists, including needle-stick injuries, biological, musculoskeletal, chemical, and psychosocial hazards, in accordance with infection control protocols, beliefs, and practices utilized by artists.

## Review

Methods 

This scoping review was conducted in accordance with the Joanna Briggs Institute (JBI) methodology for scoping reviews and the Preferred Reporting Items for Systematic reviews and Meta-Analyses extension for Scoping Reviews (PRISMA-ScR) reporting guidelines. An a priori protocol was registered on the Open Science Framework [10,11]. 

A preliminary search of MEDLINE, Google Scholar, and the Cochrane Database of Systematic Reviews was conducted on September 19th-20th, 2025, to identify existing reviews and refine search terminology. A comprehensive search strategy was subsequently developed and applied in PubMed and Ovid on September 25th, 2025, using Boolean operators and controlled vocabulary terms (see Table 1). 

**Table 1:** MEDLINE (PubMed), MEDLINE (Ovid), and Cochrane Library Search Strategies Search strategies used for the identification of studies examining occupational hazards among tattoo artists. Searches were conducted in MEDLINE (PubMed), MEDLINE (Ovid), and the Cochrane Library using combinations of controlled vocabulary terms and free-text keywords related to tattoo artists and occupational risk factors. Searches were limited to human studies published in English from 2000 to the search date. No eligible studies were identified through the Cochrane Library search.

Search Strategies
MEDLINE (PubMed): Date searched: September 19^th^, 2025 Limits: Humans: English: 2000-2025 ("tattoo artist*" OR "professional tattooist*" OR "tattoo practitioner*" OR "tattoo professional*") AND ("occupational hazard*" OR "occupational risk*" OR "workplace hazard*" OR "work-related risk*" OR "needle-stick injury*" OR "bloodborne pathogen*" OR "chemical exposure" OR "musculoskeletal disorder*" OR ergonomics OR "psychosocial stress*") Filters applied: Humans; English; Publication date from 2000/01/01 to 2025/09/19
MEDLINE (Ovid): Date Searched: 20^th^ September, 2025 tattoo artist*.mp. tattooist*.mp. body art worker*.mp. 1 OR 2 OR 3 occupational hazard*.mp. occupational risk*.mp. workplace exposure*.mp. injury*.mp. musculoskeletal disorder*.mp. bloodborne pathogen*.mp. chemical exposure*.mp. infection*.mp. Occupational Diseases/ Occupational Exposure/ Musculoskeletal Diseases/ Skin Diseases, Occupational/ Infectious Disease Transmission, Occupational/ 5 OR 6 OR 7 OR 8 OR 9 OR 10 OR 11 OR 12 OR 13 OR 14 OR 15 OR 16 OR 17 4 AND 18 limit 20 to Humans
Cochrane Library: Date searched: September 20^th^, 2025 Results: No eligible studies returned. (“tattoo artist” OR tattooist) AND (“bloodborne pathogen” OR “needle-stick” OR “musculoskeletal” OR ergonomics OR stress OR burnout OR “chemical exposure”

Additional articles were identified through citation searching and reference list review. The search was limited to articles published in English from 2000 to 2025. Books, conference abstracts without full text, and non-scholarly media (e.g., blogs, videos) were excluded.

Following the removal of duplicates, titles and abstracts were screened for relevance to the review question. Categories of risk were developed based on themes identified during article screening, including biological exposure, musculoskeletal complaints, psychosocial complaints, adverse chemical exposure, and sharp instrument injury. The criteria for inclusion based on abstract screening included the study of occupational risks to tattoo artists themselves. Exclusion criteria specifically eliminated studies focusing on the risks of those receiving tattoos, the adverse reactions of tattoos themselves, and non-professional tattoo artists, including informal, incarcerated, or amateur tattoo artists. For the purposes of this review, professional tattoo artists were defined as individuals described in the included studies as practicing tattooing in a formal commercial setting (e.g., employment in a tattoo studio or shop).

The literature on professional tattoo artists describes occupational hazards across biological, sharps-related, musculoskeletal, chemical, and psychosocial domains [4-9,12-21].

Data extraction was performed by two independent reviewers using an agreed-upon data extraction tool. The data extraction tool was developed by the authors specifically for this scoping review and was applied consistently across included studies. The tool is presented in Table 2.

**Table 2:** Characteristics of Included Studies and Occupational Hazards Among Professional Tattoo Artists This table presents the study characteristics and occupational hazards extracted from the included literature using a standardized data extraction framework. Extracted variables include author, year, and country of origin; study design and methodology; sample size and participant demographics; regulatory and licensure status; occupational hazard categories; and key findings related to occupational risk among professional tattoo artists. PPE: personal protective equipment, REBA: Rapid Entire Body Assessment, OWAS: Ovako Working Posture Analysis System, MIAR: Method for Inspection and Risk Assessment, MART: Method for Analyzing and Risk Treating, RULA: Rapid Upper Limb Assessment, MVE: maximum voluntary contraction, MSK: musculoskeletal, CTS: carpal tunnel syndrome, BBP: bloodborne pathogens

Author, year, country	Study design, methods, participant number	Demographics, licensing	Hazard type, findings
Santos et al. [20] 2024, Portugal	It is a cross-sectional workplace risk assessment comparing self-perceived risks vs formal occupational risk scoring. N=207 Ergonomic assessment tools: OWAS and REBA. General risk methods: William Fine, MIAR, and MART.	The median age is 34 years old. 66.7% male, a range of professional experience. Licensing: None. Unregulated in Portugal. No standardized training. The field is open, and training is highly variable.	Examined chemicals, sharps, biological, and musculoskeletal. Objects, contaminated blood, cleaning of skin, and ergonomic risk assessment. The most valued risks by artists were ergonomic. Sustained posture and repetitive movements were the most highly rated risks. Occupational hazard risk assessments valued chemical and biological agents more highly than artists. This effect may be due to the immediacy effect of back pain in contrast with infectious or chemical agents causing delayed harm.
Santos et al. [15] 2023, Portugal	Cross-sectional survey, descriptive observational. N = 207. Online surveys were distributed nationally, and online interviews were conducted during COVID-19. Quantitative analysis using Mann-Whitney, Kruskal-Wallis, Kendall's Tau-b, and Shapiro-Wilk for associative and correlational analysis. Qualitative interview.	Male 66.7%, ages 30-50 68.3% Portuguese nationality 91.8%, half had less than 5 years experience. Portugal does not maintain licensure.	Examined musculoskeletal, biological, and psychological risks. COVID-19 increased the adoption of PPE and standardized hygiene practices. Use of gloves and masks was high, but eye protection was rarely used. The surveys were more compliant with safety practices. The overall risk perception of infection increased. Artists with high perceived risks were more compliant with safety measures. Some safety measures were implemented voluntarily; some were due to COVID-19 restrictions.
Tamene et al. [14] 2022, Ethiopia	Cross-sectional study, a structured questionnaire examining socio-demographic characteristics, knowledge, attitudes, and practices related to infection control. N = 172	Professional artists, licensure not required in Ethiopia.	Examined biological risk factors. The participants showed the lowest scores in understanding of infection control. 61% of participants believed that wearing gloves was an appropriate substitute for handwashing. 71% believed that dry heating was an acceptable method of sterilizing needles.
Gebska-Kuczerowska et al. [13] 2021, Poland	Anonymous observational cross-sectional study, quasi-random selection of tattoo parlors. Questionnaires administer tattoo artists n = 262 beauticians = 824	Tattoo artists were majority male, and beauticians were majority female. In Poland, regulated studios require no professional licensure.	Examined biological hazards and sharps exposure. Examined exposure to hazardous waste, compliance with waste management and risk reduction procedures. Tattoo artists correctly addressed hazardous waste disposal procedures more than beauticians. (83.3% vs 44.4%) Medical waste was collected by a specialty service in 90.1% of tattoo businesses and 63.3% of beauty salons. Tattoo artists also reported correctly disposing of sharps more frequently than beauticians. (93.1% vs 68.9%) Both discarded waste into municipal trash.
Gebska-Kuczerowska et al. [12] 2020, Poland	Anonymous questionnaire survey and audits of tattoo shops in 16 provinces. Survey and observational cross-sectional study. Tattoo artists N = 233	75% male, 51% secondary education. Average age 34.9 professional tattoo artists, no associated licensure. Registered and inspected shops.	Examined biological, sharps, knowledge, attitudes, and behavior regarding blood-borne infection risk. Tattoo artists themselves were at a higher risk for infection than the clients. 18.9% of participants reported a prior sharps injury. 34.6% of artists underwent postexposure prophylaxis training. Reasons for increased risk: lack of knowledge of post-exposure prophylaxis treatment, frequency of injuries, and overall risk of needlestick injury.
Kluger N et al. [8] 2017, France	National observational self-reported internet survey. Cross-sectional observational national study of tattoo artists, n = 448. Standardized questionnaire. SPSS 19 IBM statistical analysis. Chi-squared for categorical variables. Mann-Whitney U comparing workload and occurrence of musculoskeletal symptoms.	Professional tattoo artists; no licensure required. Members of a voluntary French tattoo union. Tattooing is not a discrete, recognized profession in France and requires no formal education.	Examined musculoskeletal and chemical risks. Musculoskeletal complaints, postural pain, and pain associated with vibrating instruments. Specific breakdown of pain distribution: finger, back, and carpal tunnel. Visual complaints 41.7. 88% of pain cases endorsed finger pain. No significant risk was found in relationships to inks or chemical exposures.
Keester et al. [17] 2017, Central Ohio	Observational study and questionnaire with follow-up collection of postural and muscle activity, Questionnaire (modified Nordic Questionnaire). Used electromyography, motion monitor data acquisition software. Postural observation concurrent with EMG. Tattoo artists n = 23	An Ohio tattoo convention. 28 male, 6 female respondents.	Examined musculoskeletal risks. Twelve-month discomfort prevalence exceeded 50% in the neck, shoulders, hands/wrists, and upper and lower back (range: 53-94%). Seventy-one percent of postures evaluated during 16 h of observation had total RULA scores of 5, 6, or 7 (investigation and changes are required soon or immediately). Static muscle activity levels in the left, right, or both upper trapezius muscles in each study participant exceeded the 2-5% MVE limit recommended in the literature. Artists experienced MSK pain exacerbated by work activities. Upper extremities, lower extremities, and eyes. The trapezius EMG measurement exceeds recommended static and dynamic limits.
Kluger [16] 2015 France	Observational self-reported internet survey. Standardized questionnaire. SPSS 19 IBM statistical analysis. Chi-squared for categorical variables. Mann-Whitney U comparing workload and occurrence of musculoskeletal symptoms. N = 36 total tattooist women n = 25.	Tattooed, pregnant female tattoo artists. Members of the French Tattoo Union. France maintains no licensure.	Examined chemical and musculoskeletal risks. Pain experienced included: 52% finger pain, 72% back pain, 28% CTS, according to the responses, no malformations or other serious diseases have been diagnosed at birth or until now in living children. Pregnancy complications in heavily tattooed female tattoo artists are at the baseline population level.
Oberdorfer [19] 2004, Sydney, Australia	Randomized controlled trial. Questionnaire given to the shop owner/manager and 1 staff. Observation by trained environmental health officers observed shop procedures. Telephone interviewers asked about compliance with local regulations, self-report of use of safety equipment, and demonstration of safety practices. Tattoo shops n = 37	Individual shops surveyed, not individual artists. Regulated shops, no specific artist licensure.	Examined biological risks. Tattoo artist practices with hygiene and safety measures are increased through observation and personalized feedback for improvement. A significant difference in infection control knowledge was not found after intervention, but hygiene performance did increase hygiene performance.
Oberdorfer [18] 2003, Sydney, Australia	Observational study and survey. Findings: Knowledge gaps existed, especially in distinguishing between disinfection and sterilization concepts. Compliance was inadequate for handwashing, gloves, and sterilization techniques. Younger and less experienced tattooers were less compliant with hygiene measures. Shop owners/managers = 41. Staff members n = 21.	Study participants: Managers: 70% male, age 30-50, 40% had 10+ years in the industry. Staff: ages 25-35. No licensure required, but New South Wales guidelines are followed.	Examined biological risks. Knowledge gaps existed, especially in distinguishing between disinfection and sterilization concepts. Compliance was inadequate for handwashing, gloves, and sterilization techniques.
Raymond et al. [7] 2003, USA	Cross-sectional survey, Tattoo artists n = 61, self-administered questionnaire, and direct observation of the infection control practices of 25 tattoo artists.	Mean age: 32 years old. Average 10 years of experience. Majority male professional tattoo artists 17 regulated observed during tattooing. Eight observed were unregulated.	Examined biological and sharps risks. Most artists learned tattooing from a mentor (87%), indicating an apprenticeship educational style. Substantial prior to blood exposure risk. 37% reported prior contaminated needle injury, 54% had Hepatitis B vaccination. 24% were unvaccinated.
Lehman et al. [6] 2010, USA (Pennsylvania and Texas)	Cross-sectional survey, Tattoo artists n = 56, Assessed for compliance with 5 administrative and 10 infection control standards for reducing exposure to BBPs. 1. Develop/use a written exposure control plan. 2. Maintain an injury log to document needlesticks. 3. Offer the hepatitis B vaccine to artists when hired and document declinations. 4. Provide initial and annual follow-up BBP training for artists. 5. Prevent cross-contamination. 6. Dispose of sharps properly. 7. Use appropriate PPE. 8. Wash hands after each procedure. 9. Dispose of contaminated waste properly.	12 recruited shops. Some shops are in “regulated areas,” some in “unregulated areas.”	Examined biological and sharps risks. All shops demonstrated compliance with infection control standards, but not with administrative standards, such as maintaining an exposure control plan, offering hepatitis B vaccine, and training staff. - Compliance was 75% or higher for all infection control elements except for proper disposal of sharps (58%) and tattoo covering equipment (50%). Association-affiliated shops were 100% compliant with 7 of 10 activities, and unaffiliated shops were 100% compliant with 4 of 10 activities.
Weisman et al. [4] 2022, Israel	Comparative cross-sectional observational study. Online survey questionnaire in closed professional groups: Tattoo artists n = 114 Dental workers n = 161 Office workers n = 296 Total n = 571	Licensed dental workers. Tattoo artists' licensure is not specified.	Examined musculoskeletal risks. Tattoo artists and dental workers did not have statistically different pain patterns. These pain patterns were not as similar in office workers. Lower back, neck, and hand are the most common tattoo areas. Neck, lower back, and upper back in dental workers. Lower back, neck, and hands in office workers. Office workers are less likely to report pain in the last year than tattoo artists or dental workers.
Adams J [9] 2012, USA focused	Qualitative ethnographic study, qualitative, historically comparable research design comparing the two histories of cosmetic surgery and tattooing. Participants = none. Descriptive.	Discusses historical topics.	Examined psychosocial and biological risks. This article compares the career trajectory and cultural acceptance of cosmetic surgeons to tattoo artists based on historical and sociological data. The more hierarchical structure of cosmetic surgery helped integrate into social norms more seamlessly and more quickly than tattooing, with its more disseminated and disorganized structure. The tattoo industry would benefit from the development of professional organizations. Tattooing and cosmetic surgery practices both benefited by adopting the trappings of traditional medicine, deemed the "medical facade."
Grieshaber [5] 2017, Toronto, Canada	Cross-sectional study survey and ergonomic posture analysis. Survey performed at a tattoo convention (Cornell Musculoskeletal Discomfort Questionnaire). Tattoo artists = 79	Primarily white, 56 male, 23 female, ~6.5 years in the industry. No centralized regulatory body or licensure. National infection control guidelines exist.	Examined musculoskeletal disorders. Tattoo artists have high rates of musculoskeletal complaints, including neck, back, shoulder, and upper limbs. Perceived discomfort was higher in tattoo artists than reported in cosmetology or dentistry. High precision movements and static positions were cited as major contributing factors.
Raymond et al. [21] 2001, USA	Cross-sectional study: Tattoo artists, n = 25	No state licensure, licensure by county. Mean age 32.	Examined biological risks. All respondents believed that bloodborne pathogens could be transmitted via tattooing, and most denied that trouble or expense were barriers to infection control. Knowledge about infection transmission and control was high and was positively associated with learning about infection control from a health official. Subjects were observed implementing an average of 44 of 62 recommended procedures. Having 10 years or more experience was associated with a lower adoption of recommendations, and highlights this population as most in need of education.

Studies were classified under the sharps category if they examined attitudes, practices, or procedures involving needles or other sharp instruments. Studies were classified as chemical if they examined risks related to chemical exposure. Literature was classified as psychosocial if it described mental health, psychological, or sociological concerns within this population. The musculoskeletal category included studies examining occupational complaints related to the musculoskeletal system. Hazards related to infection risk, personal protective equipment use, or hygiene-related behaviors were classified under the biological category. No geographic restrictions were applied. 

**Figure 1:**
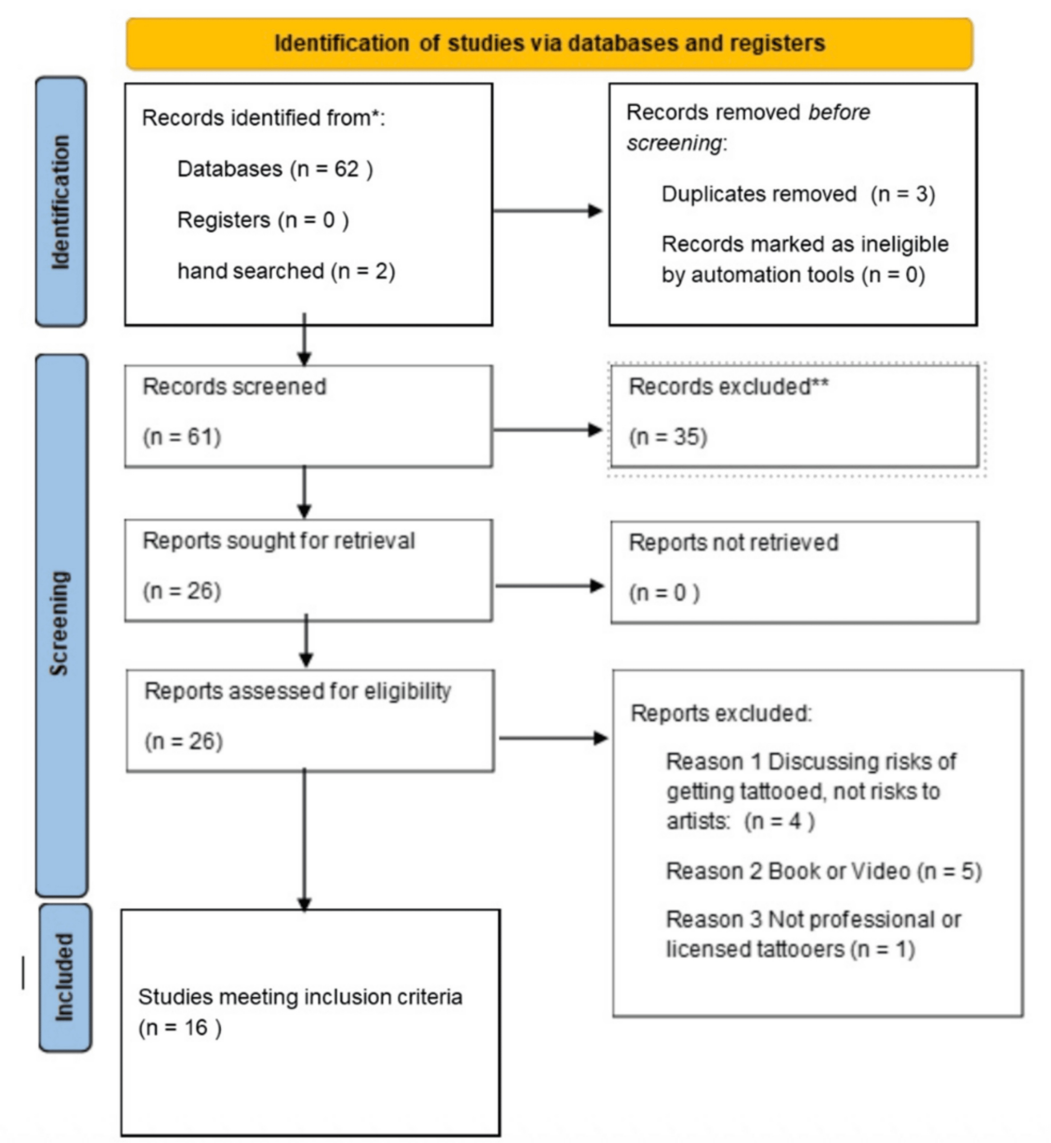
Preferred Reporting Items for Systematic reviews and Meta-Analyses extension for Scoping Reviews (PRISMA-ScR) Flow Diagram of Study Selection This figure illustrates the identification, screening, eligibility assessment, and inclusion of literature examining occupational hazards among professional tattoo artists in accordance with PRISMA-ScR guidelines. Records were identified through database searching and hand-searching of reference lists. After removal of duplicate records, titles and abstracts were screened for relevance. Records excluded at the screening stage (n = 35) were removed because they did not address occupational hazards to tattoo artists, focused on client-related outcomes, or did not meet inclusion criteria. Full-text reports were then assessed for eligibility based on predefined inclusion and exclusion criteria, with reasons for exclusion summarized. The total number of included studies is reflected in the studies meeting the inclusion criteria.

Discrepancies between reviewers were resolved through discussion between the two reviewers until a consensus was reached. Results were tabulated in PRISMA flowchart format (Figure 1). 

The included studies were analyzed qualitatively. Occupational risk factors were categorized thematically and compared across geographic region, study design, methodology, and reported outcomes. A thematic synthesis was developed to describe patterns in occupational hazards and publication trends across the literature. 

Results 

Resulting records were screened for inclusion (n=61), including those identified through database searching (n=59) and hand-searching reference lists (n=2) (Table 2). After an independent review by two reviewers, 16 records met the inclusion criteria and were included in the final analysis. Study designs overlapped between multiple studies due to mixed methodology; therefore, design counts exceed the total number of included studies. Study type was predominantly self-reported survey-based cross-sectional surveys (n=12). Observational studies (n=5) included a mixture of direct observation by regulatory bodies and researchers using validated tools. One randomized controlled trial was identified, and one qualitative descriptive analysis was identified. A subset of studies (n=4) included mixed methods.

Four studies involved a comparison between artists and other professional groups, including dental workers (n=2), workers in the beauty industry (n=2), office workers (n=1), and cosmetic surgeons (n=1).

**Figure 2:**
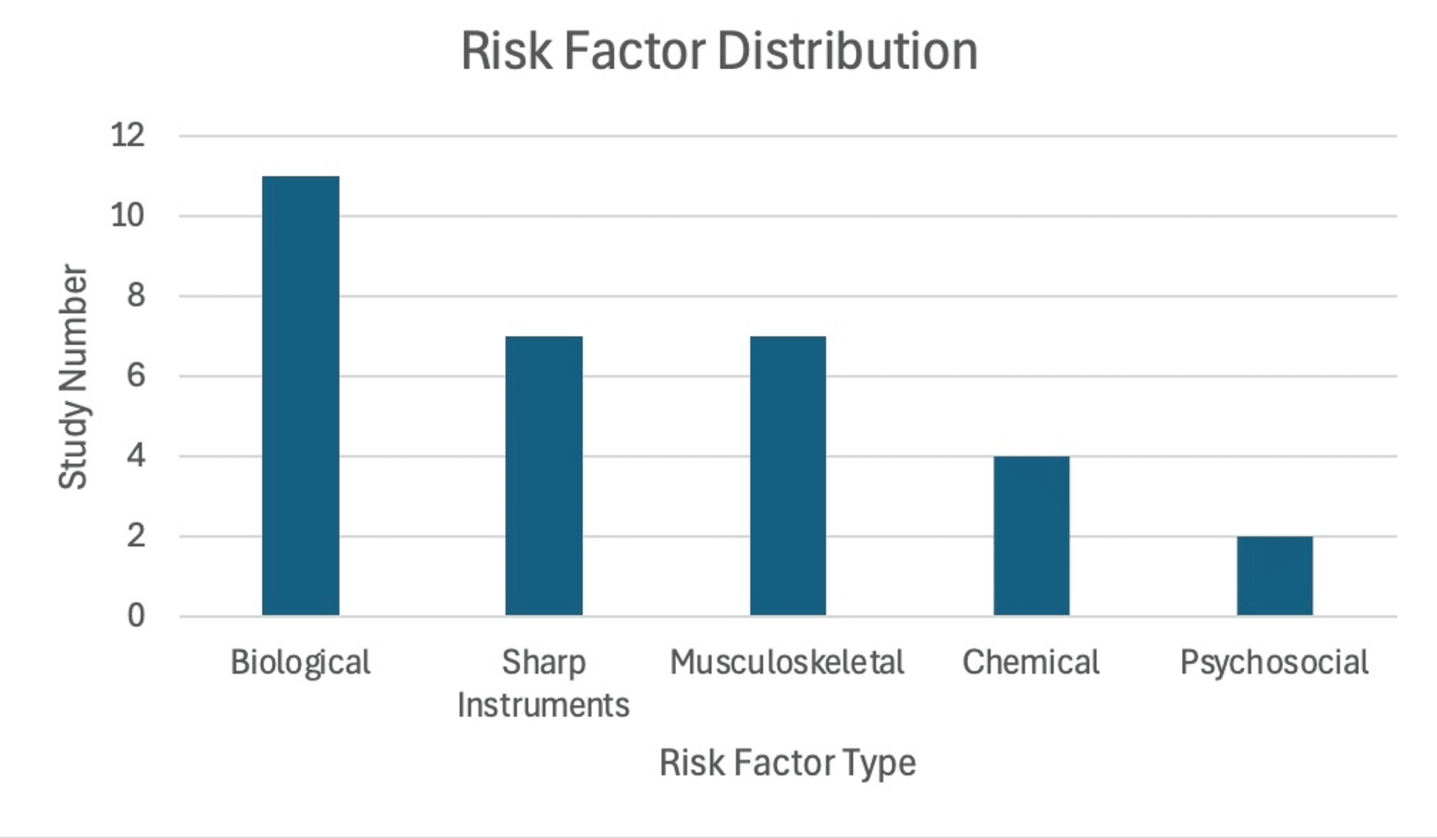
Occupational Risk Factor Distribution of Included Studies This figure illustrates the distribution of occupational hazard domains examined across the studies included in the scoping review. Several studies addressed more than one hazard category. Biological hazards were the most frequently examined, followed by sharps-related injuries and musculoskeletal disorders. Chemical and psychosocial hazards were far less studied. This figure illustrates the frequency with which occupational hazard domains (exposures, practices, and working conditions) were examined across included studies. The scoping review did not assess disease incidence or clinical outcomes but rather mapped reported occupational hazards and risk domains. The population most extensively studied is the United States (n=5). Other countries examined include Portugal (n=2), Ethiopia (n=1), Poland (n=2), France (n=2), Sydney Australia (n=2), Israel (n=1), and Canada (n=1) [4-9,12-21].

Several studies encompassed two or more domains. The largest number of studies examined biological hazards (n=11). A particularly studied area involved the understanding, attitudes, and perceptions of artists surrounding occupational safety procedures (n=7). There were seven studies examining sharp instrument injury to artists (n=7) and seven studies examining musculoskeletal harms (n=7). Of musculoskeletal concerns studied, back pain was the most studied (n=5), with neck pain (n=2), hand pain (n=3), arm pain (n=2), and eye strain (n=1) being the least studied. A prominent theme in musculoskeletal complaints was discussion of static and repetitive posture and movements contributing to pain (n=4). There were four studies examining chemical risks (n=4) and two examining psychosocial risks (n=2) (Figure 2).

The population most extensively studied is the United States (n=5). Other countries examined include Portugal (n=2), Ethiopia (n=1), Poland (n=2), France (n=2), Sydney, Australia (n=2), Israel (n=1), and Canada (n=1).

Of the selected studies, the majority were published in the last decade (n=10), and the minority were published more than 10 years ago (n=6).

Thematic summary 

Academic literature on occupational risks of tattooing remains extremely limited. Across the retrieved studies, research on occupational hazards among tattoo artists was characterized by a predominance of cross-sectional, self-report survey designs, with relatively fewer observational or experimental approaches. A paucity of experimental designs utilized standardized or validated research tools. Literature was frequently excluded in accordance with the inclusion criteria for examination of the risks of tattoos themselves, or being tattooed, and not occupational risks to artists. Biological, sharps-related, and musculoskeletal risks were the most frequently examined hazard categories, whereas chemical exposures and psychosocial risks were examined far less often in the literature. Several studies established a literary precedent for comparison of this workforce to medical proceduralist professions by comparing artists to other professional groups, including dental workers, office workers, cosmetic surgeons, and beauty-industry workers. No case reports describing occupational exposure events among tattoo artists or post-exposure prophylaxis care were identified, limiting the evidence base available to inform targeted occupational safety guidelines and medical care. The literature was geographically concentrated in North America, Europe, and Australia, with no eligible studies from South or Central America or Asia.

Discussion 

A notable finding of this review is the imbalance in research focus between (tattoo) artists and tattoo recipients. Many of the studies excluded were based on their examination of the risks of receiving tattoos and not discussing the risks of performing tattooing. Far fewer studies fit our inclusion criteria because few examined occupational risks to tattoo artists themselves. This trend was particularly pronounced in traditional medical and dermatologic journals, where client safety was emphasized more frequently than artist safety. Considering the finding that health risk may be higher to artist than clients and the relative asymmetry in group hazard exploration, this is an important area of future study.

A narrow category of study designs exists in the current literature on this topic, with the majority being cross-sectional survey-based studies. Considering the self-report nature of many of the studies, the accuracy of reporting safety-related practices could be questioned. Fears of punitive or further administrative constraints could bias a respondent towards answering questions in a more favorable way. Future research should include validated tools and objective measurements to quantify and qualify risks for the best accuracy.

Because the majority of included studies relied on self-reported survey data, the potential for recall bias and social desirability bias is high. Participants may underreport unsafe practices or environments, particularly in contexts where regulatory, legal, or reputational concerns are present. The limited availability of literature using objective measurements to examine workplace safety practices highlights the lack of attention this population has received and the utility of real-time examination of practices by future researchers.

Four studies examined the comparison of risks and risk-prevention practices between artists and other groups. These groups included dental workers, office workers, cosmetic surgeons, and beauty-industry workers. There are no case reports to date documenting post-exposure (medical care) prophylaxis care protocols for tattoo artists, like there are for medical professionals.

Notably absent from these search results are substantial discussions of the potential ethical or legal considerations of tattoo recipients' disclosure of health information in the event of a needle-stick injury to the artist. Tattoo artists do not have standardized pathways and policies to request access to medical records. This is directly contrary to common practice in medicine and related industries, wherein practitioners have the protocols and pathways to request patient consent to disclose relevant health information or perform new testing after needle-stick injury at the patient’s discretion.

A notable finding of this review is the substantial variability in tattooing regulations and licensure requirements across countries and regions. This raises an important methodological consideration: whether tattoo artists should be examined as a single occupational group, or whether region-specific studies provide more meaningful insight. Because regulatory standards, training requirements, and licensing enforcement differ widely, the generalizability of findings from one region to another is limited. The lack of medical and occupational literature on tattoo artists places individual artists at a disadvantage in receiving evidence-based medical care in the event of a needle-stick or other biological hazard encountered while tattooing.

Chemical hazard exposure to tattoo artists is an under-explored research topic. Future research should focus on this risk category, particularly given the potential for long-term impacts on artist health.

This review included literature from multiple countries as well as regional-specific studies within the United States. This geographical diversity highlights the differences in regulation and licensure across regions. Future study is needed to compare occupational risks and outcomes in differently regulated regions. Future studies should focus on direct observational or studies using objective tools to estimate occupational hazards and place them in a regional context to best inform policy and risks to individual artists. As tattooing becomes increasingly popular, it is imperative that those entering the industry are informed of potential risks for best self-protection.

Limitations 

A limitation of this review is the absence of a reliable, peer-reviewed estimate of the number of practicing tattoo artists in the United States. Because tattooing is not classified as a distinct occupational category by the U.S. Bureau of Labor Statistics and licensure is regulated at the state or local level, there is no centralized workforce registry in the United States. As a result, baseline population estimates and national exposure-risk generalizations remain unclear.

One limitation of this review is the exclusion of books and other media sources. Considering the established lack of global or national consensus on regulation and licensure, trade-specific publications may have been used to report occupational health information that is not available by traditional academic search methods.

A significant limitation of the existing literature is its geographical distribution. Absent from our results were studies from South or Central America or the continent of Asia. Tattoo culture exists worldwide, and these populations are underserved in the discussion of artist risk. In discussing the considerations of licensure, regulation, and compliance literature, this review did not evaluate the occupational requirements in countries outside of the United States in detail. A limitation of this study is conceptualizing these findings in the context of studies included in the literature that are outside of the United States regulatory agencies. Licensing and regulation are also highly geographically dependent, considering some countries were noted to have no formal licensure by the studies. Therefore, despite this review’s findings, including research from a variety of countries, the generalizability of these conclusions to countries outside of the United States is limited. Future study is needed to compare occupational risks and outcomes in differently regulated regions based on licensure and regulation.

## Conclusions

This scoping review demonstrates that, although occupational risks for tattoo artists are clearly recognized, the overall body of academic literature addressing these risks remains limited. While tattoo artists are sometimes discussed in comparison with other procedural professions, a comprehensive understanding of how to best support this workforce is lacking. Much of the existing research relies on self-report data regarding safety practices and risk awareness, with relatively few studies using objective assessment methods. Musculoskeletal strain and biological hazards are more commonly addressed, whereas chemical and psychological risks are less commonly considered. 

The literature review reflects a diverse geographical distribution. Greater emphasis on comparative and regionally contextualized research would further inform strategies to support artists. Future work would benefit from direct observations and objective measurements of workplace exposures, and case reports of medical outcomes to better characterize hazards and inform policy and medical practice.

This review highlights the need for expansion of the academic understanding of risk factors in this population. The literature base is in need of expansion with further experimental designs utilizing objective measurements and validated tools. Furthermore, it is recommended that medical literature look to document case reports of incidents of occupational harm in artists and the resulting medical management. Further understanding of this topic is needed to best support this population with policy and evidence-based medical practice. 
